# Osteoporosis in Men with Diabetes Mellitus

**DOI:** 10.4061/2011/651867

**Published:** 2011-06-15

**Authors:** Claire Issa, Mira S. Zantout, Sami T. Azar

**Affiliations:** Department of Internal Medicine, Division of Endocrinology, American University of Beirut-Medical Center, P.O Box 11-0236, Riad El Solh, Beirut 1107 2020, Lebanon

## Abstract

Osteoporosis is more common in women than in men. The prevalence in men is not defined yet; however it is becoming much more recognized as its prevalence and impact have become explicable. It is estimated that around 1% of bone mineral density is lost in men every year. Studies show that secondary osteoporosis is the major cause thus, making it important to define the disorders associated with male osteoporosis. Diabetes is a risk factor for bone fractures. In male patients with diabetes measures should be undertaken such as encouraging exercise, assuring adequate calcium and vitamin D intake, and treating diabetic complications.

## 1. Introduction

Although it is the most common cause in women, primary osteoporosis is less common in men, but it is a disease that is being more and more recognized. Prevalence has always been a challenge to determine, mainly because of the lack of consensus on the definition of osteoporosis in men. With age, men are estimated to lose around 1% of bone mineral density per year [1, 2]. Despite that, osteoporosis in men should not be assumed to be primary because some studies have shown that more than 50% of osteoporosis in men has a secondary cause [3]. The definition of secondary osteoporosis is bone loss resulting from a specific, well-defined disease. Since this form of osteoporosis can respond to the treatment of the underlying disease, and with the presence of many treatment options now available, it is imperative to recognize the disorders that are associated with male osteoporosis. More evidence is evolving about the association between diabetes and osteoporosis in both men and women. Both conditions affect a large proportion of men, thus, it is very important to assess whether there is a causal relationship that might orient further screening and management of male osteoporosis. Men and women with diabetes were found to have higher risk of fractures compared to nondiabetics [4–9]. The risk seems to be multifactorial, with osteoporosis gaining more and more interest recently. This association seems to be race, sex, and type dependent.

## 2. Type 2 DM

BMD changes in males with type 2 diabetes are very controversial, with both tendencies toward higher, normal, or lower values. In studies assessing osteoporosis in diabetic patients, osteoporosis was defined according to the WHO definition with T-score > −1 as normal, between −1 and −2.5 as osteopenia, and <−2.5 as osteoporosis. The Rotterdam study [10] was a cross-sectional study that measured BMD at lumbar spine and proximal femur using DXA in 243 DM men and 2238 healthy men. It is one of the largest studies on BMD in type 2 DM. The study showed around 3% higher BMD at both sites in DM vs non-DM subjects that remained significant even after adjustment for confounders, mainly BMI and age. 

Another study showing higher BMD in diabetic men was the EVOS study [11] that is a population-based prevalence study evaluating the effects of diabetes on bone density (measured using DXA at lumbar spine, femoral neck and femoral trochanter) and bone deformity prevalence in DM men versus non-DM. The study demonstrated that men with DM not treated with insulin had an increase in BMD only at the spine that was significant even after adjustment for body weight.

In the Health, Aging, and Body composition study [12] by Strotmeyer et al. 323 both white (38%) and black (62%) men with type 2 DM were evaluated. Fat mass and lean body mass were measured using DXA and CT. The study reported higher BMD (4-5%) at the hip in both races that was independent of body mass and composition, and the results were in concordance with older studies that also showed higher BMD in type 2 DM [13, 14].

Krakauer et al. [15] evaluated 109 diabetic patients (46 type 1 and 63 type 2). In this study, radial bone density, bone markers, and bone biopsy (in 8 patients) were assessed. It was shown that there was lower radial bone density in both groups relatively to nondiabetic controls, with no difference between patients with either type of diabetes. Transiliac bone biopsy results showed decreased bone formation and mean adjusted apposition by 75% and 70%, respectively. Some of these patients were followed up after 2.5 years (41 patients) and 12.5 years (35 patients) showing that bone loss continued at an expected rate in type 1 with maintenance of the same deficit, whereas in type 2 there was a slower than expected loss such that the initial deficit was completely corrected. 

In contrast to the above studies finding higher BMD in type 2 diabetic men, other studies showed no difference in BMD [15–17]. In one of them, Tuominen et al. [18] showed no significant difference in BMD between men with type 2 diabetes and controls at the femoral neck and trochanter. The study involved 56 patients with type 1 DM and 68 patients with type 2 DM from both sexes along with 498 non-DM controls. Similar findings were shown in a study by Shwartz et al. [19] evaluating bone loss at the hip over 4 years (measuring BMD at baseline and at the end of 4 years) in 480 DM men and women, 439 with impaired glucose metabolism, and 1172 healthy controls. It was found that despite having higher baseline BMD, only diabetic white women, but not black women nor men with DM and impaired glucose metabolism, demonstrated significant bone loss.

Other studies, on the other hand, showed lower BMD in patients with type 2 DM [20, 21]. In a cross-sectional study [22] involving 735 type 2 DM and 3458 nondiabetic men, BMD at the hip and spine was measured. This study showed lower BMD at the hip and higher incidence of osteoporosis in diabetic men that was significant even after adjustment for age and BMI. BMD at the spine was significantly higher in diabetics when compared to controls, but when adjusted for BMI, it became similar. 

In another study by Petit et al. [23], using peripheral quantitative CT (pQCT) this time instead of assessing BMD by DXA to measure tibial and radial bone volumetric density, bone geometry, and bone strength. Bone strength was determined by measuring estimates of bone compressive and bending strength. Calculated bone strength index was used as an index of bone compressive strength, and calculated strength strain index was used as an index of bone bending strength. It was shown that older men with type 2 DM have bone strength that is low relative to body weight at the cortical-rich midshaft of the radius despite no difference in cortical bone volumetric density. This can account for the increased risk of fractures despite higher BMD in type 2 DM patients which might incriminate DXA as being a weak tool to assess bone in type 2 DM males.

A conclusion is very hard to draw after all the controversies shown in those studies, and to make things even more complicated, all those trials neglected to study the cortical bone which is a major limitation since bone is heterogenous (cortical and trabecular) and since diabetes, as many other endocrinological disorders such as hyperparathyroidism and hyperthyroidism, might affect cortical bone more than trabecular bone.

In order to correct for this limitation, other studies tried to study the BMD at the cortical bone showing that diabetes can actually affect bone heterogeneously by affecting cortical more than the trabecular bone [24, 25]. One of them [25] was conducted on 64 diabetic and 41 healthy Japanese men. BMD was measured using DXA at the lumbar spine, femoral neck, and distal radius, and it showed a significantly lower BMD at the distal radius in type 2 DM patients versus controls, that was even lower than their own BMD at the spine and femur. In type 2 DM, there was a negative correlation between BMD at the distal radius and mean HBA1C during the past 2 years. These findings demonstrate the importance of measuring 3 sites in patients with type 2 DM because of the possible selective cortical involvement.

Since diabetes is preceded by several years of pre-diabetic stage, it is worth to study the effect of impaired fasting glucose (defined as fasting glucose between 100–125 mg/dL) or impaired glucose tolerance (defined as a 2 hr glucose level between 140–200 mg/dL post 75 gr oral glucose load) on the BMD, in order to try to find a pathophysiology behind osteoporosis in diabetes and to try to find when does the effect on BMD start and whether there is a way to prevent it or stop it. Unfortunately, few studies evaluated BMD in prediabetic men. One of them [26] compared BMD in 272 men with prediabetes and 406 normal men. The study showed no difference in BMD between the 2 groups. However, when the prediabetic men were divided into quartiles based on fasting insulin and insulin levels 2 hrs after-75 gr glucose, it was noted that the BMD T-score increased with the increase in fasting insulin (*P* = .004). Additionally, the subjects with the highest concentrations of fasting insulin belonged to the groups with higher BMD T- scores (*P* < .001).

## 3. Type 1 DM

As in type 2 DM, there is also some controversy as to the association between type 1 DM and osteoporosis, but in contrast to type 2 DM most [27–31] but not all [32, 33] studies showed decreased BMD. In contrast to type 2 DM, there seems to be an important gender difference with more marked bone loss in men versus women when compared to matched controls [27–30].

 The same study by Tuominen et al. [18] on both type 1 and type 2 DM patients of both genders, measuring BMD using DXA at the proximal femur, revealed that among both sexes, BMD values are significantly lower in type 1 versus type 2 DM or controls. The difference between type 1 DM and controls remained significant in both sexes even after adjustment for age and BMI, whereas the difference between type 1 and type 2 remained only significant in men. The latter difference remained unaltered after further adjustment for duration of diabetes, but was slightly reduced when additionally adjusted for duration of insulin treatment and dose.

In another study [28] conducted on 30 type 1 DM men and 30 type 1 DM women versus 60 healthy controls, followed retrospectively, it was shown that male patients with type 1 diabetes has a significantly lower BMD values and lower Z-scores at the spine and femoral neck when compared with healthy men (*P* < .05). This difference remained the same after adjustment for age. The percentages of both osteoporosis and osteopenia were higher in DM men when compared to both normal men and diabetic women. There was no significant correlation between age-adjusted BMD values, and either diabetes duration, HBA1C values or age of onset of diabetes. Femoral neck BMD values were positively correlated with BMI in both female groups but only in healthy men. In conclusion, this study showed low BMD values in type 1 DM men and showed the gender difference on the effect of diabetes on BMD where diabetic men had lower BMD values when compared with diabetic women.

Few studies have assessed bone markers in diabetic men. In one of them [31], both BMD (measured by DXA) and serum bone markers (osteocalcin, C-terminal telopeptide of type 1 collagen (CTX), leptin and osteoprotegerin (OP)) were measured in 42 adult type 1 DM men and 24 non-diabetic controls. It was shown that 40% of type 1 DM patients had osteopenia at the spine and/or hip and 7% met criteria for osteoporosis. BMD z-score was correlated with age, negatively correlated with CTX, and osteocalcin. Osteocalcin, CTX and leptin concentrations were comparable in both groups, while OPG concentrations tended to be higher in DM. Despite the fact that there was not an increase in bone resorption markers in this study, this does not exclude a previous state of increased bone resorption. This is favored by the increase in OPG observed in this study that can be a protective mechanism of the skeleton to compensate for the possible previous increased bone resorption and bone loss.

In another study, where more bone markers and hormonal markers, especially testosterone, were measured, Hamilton et al. [33] conducted a cross-sectional trial involving 50 type 1 DM men, and 50 healthy controls, aged 30–71 years, assessing biochemical/hormonal markers of bone metabolism (25-hydroxy vitamin D3, PTH, CTX, osteocalcin, procollagen type 1 N-terminal propeptide (P1NP), total and free serum testosterone, sex hormone-binding globulin (SHBG), luteinizing hormone (LH), and follicule stimulating hormone (FSH)). BMD values at forearm, spine, and hip were recorded. It was shown, after adjustment for age and BMI, that BMD values, T and Z-scores were lower in DM men versus controls (*P* < .048). Prevalence of osteoporosis and osteopenia was higher only at the spine in DM men (*P* = .03). After adjusting for age and BMI, the investigators found that BMD was not significantly associated with HBA1C, smoking, nephropathy, retinopathy, or neuropathy. Only higher BMD at the spine was associated with diabetes duration. On multiple linear regression analysis, which adjusted for the natural logarithm (Ln) of the sex hormone-binding globulin concentration, smoking status, and alcohol consumption, it was shown that serum alkaline phosphatase was significantly negatively associated with BMD at 3 sites. There was a positive association between Ln (free testosterone) and BMD at the forearm and a negative association between Ln (osteocalcin) and BMD at the forearm. 

In a Dutch study [34], osteopenia was found in as many as 67% and osteoporosis was seen in as many as 14% of 21 type 1 DM men compared to healthy controls. It was shown also that osteopenia was associated with low serum IGF-1 levels and bone formation markers.

In the study that was mentioned previously, conducted by Krakauer et al. [15], again it was shown that radial bone density was lower in type 1 DM relatively to nondiabetic controls, but this low BMD seems to be stable over time, with expected stable bone loss, according to this study.

In another study [35], trying to follow type 1 DM patients over time, to study for the changes in bone density, 41 type 1 DM (19 female, 22 males), mean age 9 years, were followed for around 5 years. Two sites of the nondominant radius were analyzed by pQCT. At the distal radius (metaphyseal site) total and trabecular BMD and at the proximal radius (diaphyseal site) total and cortical BMD, total cross-sectional area (CSA), cortical CSA, medullary CSA, muscle CSA, and strength strain index as measure of bone stability were calculated. It was shown in this study that at the 1st evaluation, mean SD value of trabecular BMD was even higher in type 1 diabetic patients than in controls, irrespective of age, sex, and Tanner stage. At the diaphysis, patients with type 1 DM had significantly reduced mean SD values for total, cortical, and medullary SCA as well as cortical BMD which had normalized at the 2nd measurement. The younger the patients were at the disease manifestation and at the 1st evaluation, the more the increase in total CSA was detectable. As a conclusion, this study suggests a defect in bone accretion early in the course of type 1 DM, which then ameliorates with time. A limitation of this study was the small number, but the strength is using pQCT as a tool for bone measurements and the 5-year followup.

Moreover, in another study [36] using both pQCT and DXA to measure bone mass and structure in 48 adolescents with type 1 DM (26 girls and 22 boys), pQCT measurements were performed at the distal and shaft sites of the dominant radius and the right tibia. BMC, total cross-sectional area and trabecular density were determined for the distal sites, BMC, cortical density, and cortical cross-sectional area were determined for the shaft sites. It was shown that diabetes was associated with reduced bone mineral content (BMC) and smaller bone cross-sectional size, with boys being more affected than girls with a mean deficit in BMC of all measured skeletal sites of >10% in boys and <5% in girls.

## 4. Type 1 and Type 2 DM

Because most of the studies involved a small number of patients, in order to correct for this limitation and increase the power, a meta-analysis [30] including 80 papers on BMD and fracture risk in patients with type 1 and 2 DM was done and showed that in both genders there was an increased risk of fractures in both types of diabetes mellitus compared to non-diabetes mellitus. Z-score in hip and spine was decreased in type 1 and increased in type 2 DM. A meta-regression showed that mainly BMI was the major determinant for BMD, whereas HBA1C was not linked to BMD. The increase in fracture risk was higher and BMD was lower in patients with complications of DM.

### 4.1. In Conclusion

The relationship between diabetes, both types, and osteoporosis seems complex. Mainly because the studies were limited by the small number, patients werenot followed up for a long period of time, studies were heteregenous with different diabetes control and duration, different complications, which makes it hard to draw a unique conclusion. But the trend seems to be a low BMD in type 1 DM and a high BMD in type 2 DM. More studies correcting for the mentioned limitations need to be performed.

## 5. Pathophysiology of Altered BMD in Diabetic Patients

Several hypotheses have been raised to explain the altered BMD in diabetic patients. Although the most popular one was the higher BMI in type 2 DM that can protect from osteoporosis, most of the studies discussed previously corrected for BMI, yet despite this correction there was still a higher BMD in patients with type 2 diabetes relatively to type 1 and healthy controls.

## 6. Insulin and Insulin Growth Factors

One popular hypothesis to the effect of diabetes on bone is through insulin, acting as a bone anabolic factor. The importance of this hypothesis is that it can also explain the pathophysiology behind the difference between type 1 and type 2 diabetic men. Insulin seems to have both direct and indirect effects on bone.

Support for a direct role of insulin in bone comes mainly from animal studies, specifically rats, where streptozocin-induced diabetes led to defects of bone mineralization. Moreover, rats lacking insulin receptors had impaired bone formation and low bone turnover [37–39]. In a recent study conducted by Fulzele et al. [40], to try and directly examine the function of insulin signaling in bone, they engineered mice lacking insulin receptor (IR) specifically in osteoblasts. It was shown that osteoblasts lacking IR had severely impaired differentiation, with increased apoptosis. They showed a 79% decrease in the number of osteoblasts per bone perimeter at 3 weeks of age. This led to a dramatic impairement in postnatal trabecular bone acquisition. Administration of IGF-1 did not correct for the abnormalities seen, showing the direct effect of insulin, independently of IGF-1 on the bone. A high level of expression of insulin receptors on osteoblasts was reported [41], and insulin binding to these receptors led to cell proliferation, production of alkaline phosphatase, collagen synthesis, and glucose uptake [42–45]. The effect of insulin seems not only limited to osteoblasts, because in vitro studies showed that osteoclasts as well have insulin receptors where insulin can act to inhibit their action [46]. 

Human data support this hypothesis. A large study comprising of over 100 subjects with type 1 diabetes mellitus showed lower IGF-1 levels as well as bone formation markers when compared to healthy controls [47, 48]. Moreover, low levels of IGF-1 were found to be associated with osteopenia in type 1 DM patients [48].

 In contrast to type 1 diabetes mellitus where there is insulin deficiency, in type 2 diabetes mellitus there is insulin resistance and hyperinsulinemia which can explain the higher BMD in type 2 diabetes mellitus. Since the insulin resistance is selective and only restricted to the effect of insulin on glucose transport [49], the high insulin levels can still act on the osteoblast to increase BMD. Indeed some investigators found a positive correlation between insulin levels and BMD [50, 51], yet others did not [52, 53].

In addition to a direct effect of insulin on osteoblast and osteoclast, insulin can indirectly act on the bone by decreasing sex-hormone binding globulin [54–57] leading to higher levels of free estrogen and testosterone, acting positively on the bone to increase BMD [58]. It can act indirectly by suppressing IGFBP-1 thus increasing the sensitivity of osteoblasts to IGF-1, then IGF-1 will modulate the actions of PTH on bone leading to a synergistic effect between insulin and PTH as well an indirect synergistic effect with other substances that mediate anabolic effects on bone [59, 60]. These are all theories, and more studies are needed to assess the effect of insulin on bone in male patients with diabetes.

## 7. Diabetic Complications

Uncontrolled diabetes with hyperglycemia has been suggested as a possible mechanism for osteoporosis in both type 1 and type 2. This can occur by the formation of nonenzymatic glycosylation of various bone proteins, including type 1 collagen, leading to impaired bone quality [61]. There are also some studies [62] associating high levels of pentosidine to higher risk of fractures in diabetic patients. Moreover, glucose is the principle source of energy for osteoclasts and is able to increase avian osteoclast activity in vitro in a dose-dependent manner [63]. Another indirect effect of hyperglycemia on bone can be through hypercalciuria secondary to glycosuria and other interactions with PTH and vitamin D metabolism [64]. Some studies have shown that type 2 diabetes mellitus is associated with lower vitamin D levels compared to healthy controls [65, 66]. Despite these theories, only some [67] but not all [33, 68] studies have demonstrated that glycemic control and HBA1C levels were associated with osteoporosis in diabetic patients.

Other than glycemic control, diabetic complications were incriminated in osteoporosis, mainly retinopathy [69–71] by decreasing exercise thus leading to decreased muscle mass and poor vision leading to increased incidence of falls, nephropathy [70, 72, 73] by affecting bone metabolism, microangiopathy by directly affecting bone vascularisation, and neuropathy [74] by decreasing exercise. Yet again other studies have shown contradictory results [28, 33] with no association between either complication and BMD.

## 8. Bone Turnover and Bone Stiffness

Most studies assessing bone turnover in diabetic patients were limited by the small number of patients included. In general, it was suggested that there is an imbalance between bone formation and bone resorption in diabetes. Kemink et al. reported a lower BMD in diabetic patients who concomitantly had lower alkaline phosphatase levels [34]. Dobnig et al. [75] showed that subjects with type 2 diabetes mellitus had lower levels of PTH and osteocalcin. The same was shown by Achemelal et al. [76]. Thus, it seems that in type 2 DM, there is a decreased bone formation. This was also shown by the study, previously mentioned above, by Krakauer et al. [15], where they came up with a conclusion that can explain the discrepancy between type 1 and type 2 DM, that diabetes is accompanied by low bone formation rate, that can lead to osteopenia in the growing skeleton, whereas in type 2 DM, there is low bone turnover that will retard bone loss, explaining the increased bone density in type 2 DM. Another contradictory study, done by Alexopulou et al. [27], showed that there was no difference in osteocalcin levels or C-terminal telopeptide type 1 collagen between males with type 1 diabetes mellitus and controls, but osteoprotegerin was increased, showing contrary to previous studies that bone formation was normal. In contrast to bone formation, most studies [27, 75, 76] have shown normal bone resorption markers in diabetic patients, suggesting with the associated low bone formation a state of low bone turnover or adynamic bone in diabetes. Again this is just a theory and more advanced studies are needed with more assessment of bone markers for both bone formation and resorption.

## 9. Hormonal Imbalance

Since hypogonadism in men was associated with low BMD and fractures, and since most diabetic men have lower testosterone levels when compared to nondiabetics, it was suggested that diabetes can cause low BMD and higher risk of fractures through causing hypogonadism. Studies on this association are very limited, with one conducted by Asano et al. [68] showing a weak but a significantly positive correlation between serum bioavailable testosterone and bone stiffness in type 2 DM.

## 10. Conclusion

Both osteoporosis and diabetes are increasing in men. Evidence of an association between both diseases is increasing, but in face of the controversy and the limitation of the studies done (small studies, cross-sectional, no clear assessment of the pathophysiology), more studies correcting for these limitations (with a longer followup, assessing bone markers, hormonal factors, complications, shifting to a better imaging, as CT&MRI instead of DXA assessment of BMD) are still needed. Despite the fact that specific evidence-based recommendations based on the present data are not available and cannot be made, there must be awareness about the fact that diabetes, especially type 1, might be a risk factor for osteoporosis, that it is multifactorial, possibly affecting cortical and trabecular bone differently. Specific measures should be undertaken as encouraging exercise, assuring adequate calcium and vitamin D intake, and treating diabetic complications.

##  Evidence Acquisition

The major source of data acquisition included Medline search strategies, using the words “type 1 diabetes mellitus,” “type 2 diabetes mellitus,” “osteoporosis,” and “men.” Articles published in the last 13 years were screened.
